# Anterior Prefrontal Hemodynamic Connectivity in Conscious 3- to 7-Year-Old Children with Typical Development and Autism Spectrum Disorder

**DOI:** 10.1371/journal.pone.0056087

**Published:** 2013-02-13

**Authors:** Mitsuru Kikuchi, Yuko Yoshimura, Kiyomi Shitamichi, Sanae Ueno, Hirotoshi Hiraishi, Toshio Munesue, Tetsu Hirosawa, Yasuki Ono, Tsunehisa Tsubokawa, Yoshihiro Inoue, Manabu Oi, Yo Niida, Gerard B. Remijn, Tsutomu Takahashi, Michio Suzuki, Haruhiro Higashida, Yoshio Minabe

**Affiliations:** 1 Research Center for Child Mental Development, Kanazawa University, Kanazawa, Japan; 2 Department of Psychiatry and Neurobiology, Graduate School of Medical Science, Kanazawa University, Kanazawa, Japan; 3 Higher Brain Functions & Autism Research, Department of Child Development, United Graduate School of Child Development, Osaka University, Kanazawa University, Hamamatsu University School of Medicine, Chiba University and University of Fukui, Osaka University, Osaka, Japan; 4 International Education Center, Kyushu University, Fukuoka, Japan; 5 Department of Anesthesiology, Graduate School of Medical Science, Kanazawa University, Kanazawa, Japan; 6 R & D Department, Medical Systems Division, Shimadzu Corporation, Kyoto, Japan; 7 Department of Neuropsychiatry, University of Toyama, Toyama, Japan; Rikagaku Kenkyūsho Brain Science Institute, Japan

## Abstract

Socio-communicative impairments are salient features of autism spectrum disorder (ASD) from a young age. The anterior prefrontal cortex (aPFC), or Brodmann area 10, is a key processing area for social function, and atypical development of this area is thought to play a role in the social deficits in ASD. It is important to understand these brain functions in developing children with ASD. However, these brain functions have not yet been well described under conscious conditions in young children with ASD. In the present study, we focused on the brain hemodynamic functional connectivity between the right and the left aPFC in children with ASD and typically developing (TD) children and investigated whether there was a correlation between this connectivity and social ability. Brain hemodynamic fluctuations were measured non-invasively by near-infrared spectroscopy (NIRS) in 3- to 7-year-old children with ASD (n = 15) and gender- and age-matched TD children (n = 15). The functional connectivity between the right and the left aPFC was assessed by measuring the coherence for low-frequency spontaneous fluctuations (0.01 – 0.10 Hz) during a narrated picture-card show. Coherence analysis demonstrated that children with ASD had a significantly higher inter-hemispheric connectivity with 0.02-Hz fluctuations, whereas a power analysis did not demonstrate significant differences between the two groups in terms of low frequency fluctuations (0.01 – 0.10 Hz). This aberrant higher connectivity in children with ASD was positively correlated with the severity of social deficit, as scored with the Autism Diagnostic Observation Schedule. This is the first study to demonstrate aberrant brain functional connectivity between the right and the left aPFC under conscious conditions in young children with ASD.

## Introduction

The importance of the anterior prefrontal cortex (aPFC), or Brodmann area10 (BA10), for higher-order cognitive functions is largely undisputed [1]–[3], and several authors have placed this brain region at the top of the frontal processing hierarchy [4]–[5]. BA10 has been implicated in the ability to infer another person’s feelings and thoughts, often referred to as theory of mind (TOM), as demonstrated by a lesion study [6] and neuroimaging studies [7]–[8]. In addition, BA10 has been implicated in the ability to check, monitor, or follow the focus of attention of the other, often referred to as joint attention, as demonstrated by neuroimaging studies [9]–[10]. In humans, BA10 is thought to be involved in the expression of sociality and appropriate social conduct.

Autism spectrum disorders (ASDs) are characterized by impairments in reciprocal social interaction and communication and the presence of stereotyped or repetitive behaviors [11]. ASDs are typically diagnosed in early childhood and expressed throughout life. Deficits in social cognition (e.g., impairment in mentalizing, TOM and joint attention) have been reported in patients with ASD [12]–[16]. Intriguingly, several neuroanatomical and regional cerebral blood flow studies on ASD subjects have found abnormalities in BA10 [17]–[19]. This finding suggests that brain dysfunction in BA10 is implicated in the impairments in reciprocal social interaction observed in ASD. However, the role of brain dysfunction BA10 in young children with ASD remains unclear.

Children with ASD present with an aberrant age-related brain growth trajectory in the frontal area [20]–[22], which strongly suggests that brain functional measurements at young ages are crucial for revealing ongoing abnormalities in children with ASD. However, it is a challenge to perform brain hemodynamic measurements using a functional magnetic resonance imaging (fMRI) on young children who are conscious because it is difficult to maintain their cooperation in experimental environments. A few studies have successfully measured aberrant brain hemodynamic responses [23]–[24] or aberrant brain hemodynamic connectivity [25] in young children with ASD under sleeping conditions. However, no studies have investigated brain hemodynamic connectivity in young children with ASD under conscious conditions. Near-infrared spectroscopy (NIRS) non-invasively and easily measures changes in cerebral blood flow, even in young children under conscious conditions. NIRS has advantages over fMRI methods, including improved safety, fewer constraints, less environmental noise and less sensitivity to head motion. NIRS is valuable for brain functional monitoring in young children under conscious conditions [26]–[27], which is difficult to achieve with fMRI. In the present study, we measured brain hemodynamic fluctuation in bilateral BA10 using NIRS in 3- to 7-year-old ASD and typically developing (TD) children under conscious conditions. To investigate hemodynamic connectivity in bilateral BA10, we employed coherence analysis to investigate connectivity in slow, spontaneous hemodynamic fluctuations (e.g., 0.01–0.10-Hz fluctuations). We focused on slow hemodynamic fluctuations because recent studies on these fluctuations have uncovered aberrant functional connectivity in adults with ASD [28]–[32].

## Materials and Methods

### Ethics Statement

**Figure 1:**
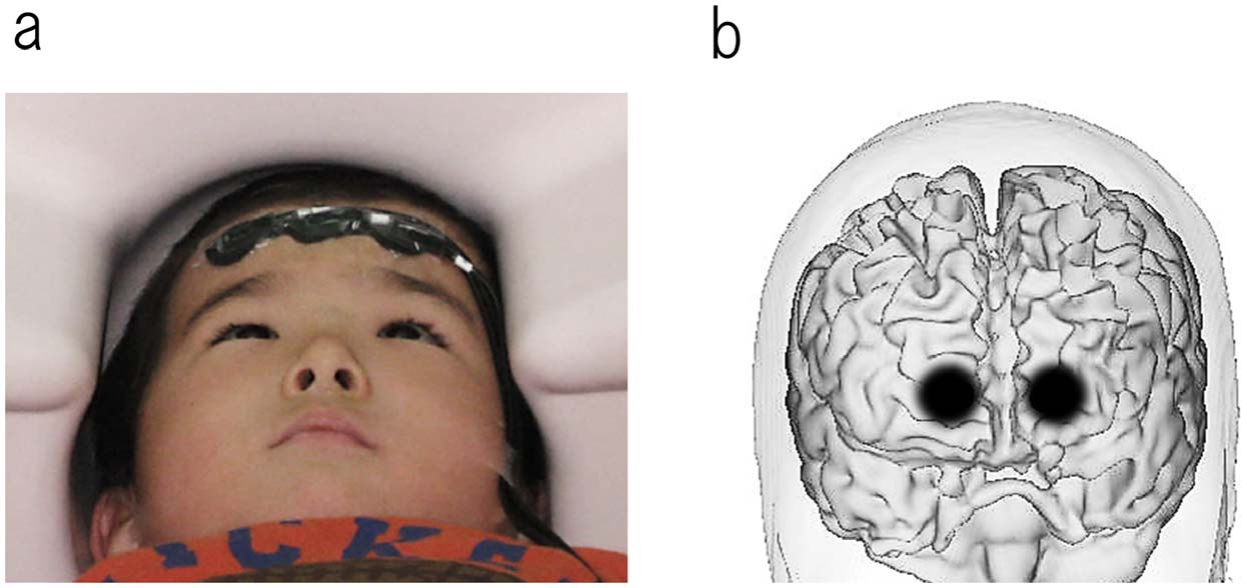
The locations of the NIRS optodes on the skull. (a) To limit movements, children were in the supine position on the bed and placed their head on the headrest. The optode set was stabilized on the participant’s forehead with an adhesive tape. (b) The locations of the NIRS optodes were determined using the international 10–20 system for EEG. The set of 3 optode probes was placed on the participant’s forehead such that the center optode probe was located on the Fpz with the optode row extending collinear along the Fp1-Fpz-Fp2 line. The darker shaded area of the brain indicates the position of the light guide for the NIRS recording in one representative.

Parents agreed to their child’s participation in the study with full knowledge of the experimental nature of the research. Written informed consent was obtained prior to participation. The Ethics Committee of Kanazawa University Hospital approved the methods, and all procedures were performed in accordance with the Declaration of Helsinki. Legal Guardian of the subject in the photograph (Fig. 1) has given written informed consent, as outlined in the PLOS consent form, to publication of their photograph.

### Subjects

The clinical group included 15 children with autism spectrum disorder (13 males and 2 females), aged 47–86 months, who were recruited from the Kanazawa University Hospital and prefectural hospitals in Toyama. Children were diagnosed by a clinical psychiatrist and a clinical psychologist with over 5 years of experience in ASD using the Autism Diagnostic Observational Schedule, Generic (ADOS) [33], Diagnostic Interview for Social and Communication Disorders (DISCO) [34], and DSM-IV criteria at the time of NIRS data acquisition. All children with ASD fulfilled the diagnosis of childhood autism (n = 10), atypical autism (n = 1) or Asperger’s syndrome (n = 4) with DISCO. Children below the ADOS cut-offs were included in the present study if they met the criteria for ASD using both the DSM-IV and DISCO (2 of the 15 children scored one or two points below the ADOS cut-off). The controls were 15 typically developing children (13 males and 2 females), aged 45–82 months, with no reported behavioral or language problems. The control children were approximately matched to the subjects with ASD by age. All TD children were native Japanese and had no previous or existing developmental, learning, or behavioral problems according to information obtained by a questionnaire that was completed by their parents. All participants had normal hearing ability according to available medical records. The dominant hand was determined by the children’s preference when handling a spoon; most of the children were right handed (TD children: right = 15, left = 0, both = 0; children with ASD: right = 10, left = 1, both = 4).

All children completed the Kaufman Assessment Battery for Children (K-ABC) [35]. The mean quotient of the K-ABC mental processing scale was 94.1 ± 17.3 (mean ± SD) for children with ASD and 99.7 ± 10.3 for TD children. The mean quotient of the K-ABC achievement scale was 90.9±20.8 for children with ASD and 100.0±13.2 for TD children. As shown in Table 1, the two groups were approximately matched according to chronological age, and there was no significant difference between the groups in terms of the mental processing or achievement quotients.

**Table 1:** Demographic characteristics of all participants.

Group	ASD children	TD children	t	p
Number of subjects	15	15		
Gender (M/F)	13/2	13/2		
Age in months	45–82	47–86	−1.92	n.s.
K-ABC mental processing quotient (± SD)	99.7±10.3	94.1±17.3	1.07	n.s.
K-ABC achievement quotient (± SD)	90.9±20.8	100.0±13.2	1.43	n.s.

K-ABC, Kaufman Assessment Battery for Children; TD, typically developing; ASD, Autism Spectrum Disorder. n.s., no significance (p>0.05).

### Conscious Conditions during Measurement

To minimize head movement, children lay on the bed in the supine position and placed their heads onto a headrest (Fig. 1a). An examiner remained in the room throughout the measurements to encourage the children and prevent movement. To detect the brain hemodynamic connectivity in the frequency range 0.01–0.10 Hz [36] with a spectral resolution of 0.01 Hz, a 100-second continuous artifact-free period was necessary for each segment in order to calculate coherence values. At first, we attempted to record during stimulation-free quiet conditions. However, the 3- to 4-year-old children had difficulty remaining still for several 100-second periods because of boredom under these stimulation-free conditions. Therefore, we altered the conditions. To maintain motion-noise-free but conscious conditions for a 100-second continuous period, we used the attractive picture-card show “Mr. Crow’s Bakery” (by Satoshi Kako), which is a popular picture-card show among young children in Japan. During the measurement, the children watched a narrated picture-card show that was projected onto a screen (for up to 20 minutes). Although their spontaneous attention was engaged in a picture-card show, six 100-second continuous artifact-free segments were obtained during the 20-minute task-free period.

### Near-infrared Spectroscopy Measurements, Equipment and Data Analysis

While the participant watched the picture-card show, NIRS measurements were made with a set of 1×3 optode probes on a continuous wave system (Foire 3000, Shimadzu Kyoto, Japan). We employed thin (5 mm) and lightweight optode probes to minimize feelings of strangeness (Fig. 1a) [37] (this is a custom-developed system that is especially important for young children). The optode set (Fig. 1a) consisted of 2 light emitters and 1 photo detector that was placed with an interoptode distance of 3 cm, which comprised 2 channels in total. Oxygenated hemoglobin (oxy-Hb), deoxygenated hemoglobin (deoxy-Hb) and total hemoglobin values were obtained, and oxy- and deoxy-Hb values were analyzed. Previous studies with adult participants have indicated a measurable depth with the interoptode distance of 15–25 mm beneath the scalp [38]. NIRS measurements were made for 20 minutes over the participants’ right and left anterior prefrontal areas with a sampling rate of 10 Hz. Following the international 10–20 system for EEG [39], the set of 2 channels (3 optode probes) was placed on the participants’ foreheads such that the optode probe of the center was located at the Fpz with the optode row extending collinear to the Fp1-Fpz-Fp2 line (Fig. 1a, 1b). An off-line analysis of the NIRS data was performed with Brain Vision Analyzer (Brain Products) and Matlab (MathWorks). With a visual inspection, data were divided into 100-second artifact-free segments. Data contaminants (primarily motion artifacts) were eliminated while the analyst remained blinded to the personal data. Six artifact-free 100-second (i.e., continuous for 100 seconds) segments (600 seconds total) were analyzed for each subject. Hemodynamic spectra were calculated with the fast Fourier transform with a spectral resolution of 0.01 Hz. We focused on the frequency range 0.01–0.10 Hz for the following reasons: brain intrinsic hemodynamic connectivity can be predominantly detected in this range [36], and this frequency range is outside the typical respiratory artifact (0.1 to 0.5 Hz) and cardiac beat (0.6 to 1.2 Hz). Coherence and absolute power values were obtained separately for 10 frequency points, from 0.01 to 0.10 Hz, for oxy- and deoxy-Hb. Coherences (Cross-Spectrum/Autospectrum) were calculated after a Fourier transform using the formula: Coherence (c1, c2)(f) = | CS(c1, c2)(f) |^2^/(| CS(c1, c1)(f) | | CS(c2, c2)(f) | ), in conjunction with CS(c1, c2)(f) = Σ c1, i (f) c2, i (f)*. In these formulas, CS represents Cross-Spectrum. In the second formula, totaling is carried out via the segment number i. Formation of the average also relates to segments with a fixed frequency f and a fixed channel c. In this method, values between 0 and 1 were obtained for each frequency and each channel.

### Statistical Analysis

Based on a recent review of functional connectivity magnetic resonance imaging (fcMRI) studies in adults with ASD [29], we hypothesized that young children with ASD would have aberrant spontaneous brain connectivity in 0.01–0.10 Hz hemodynamic fluctuation under conscious conditions. We explored the 0.01–0.10 Hz frequencies of hemodynamic fluctuation to determine which frequencies contribute to aberrant functional connectivity in young children with ASD. Unpaired *t*-tests were performed to compare the ASD and TD groups for inter-hemispheric coherence and log-transformed absolute power values in both hemispheres. Because of the multiple comparisons in 10 frequency bands, the alpha level was adjusted to 0.005 (0.05/10). When significant differences were found for the inter-hemispheric coherence or log-transformed absolute power values, Pearson’s correlation was used to determine significant correlations between physiological measures and social deficit, as scored by ADOS (summation of communication domain, the social interaction domain scores), in children with ASD. For this correlation analysis, the alpha level was set at 0.05.

## Results

### Absolute Hemodynamic Power Value

**Figure 2:**
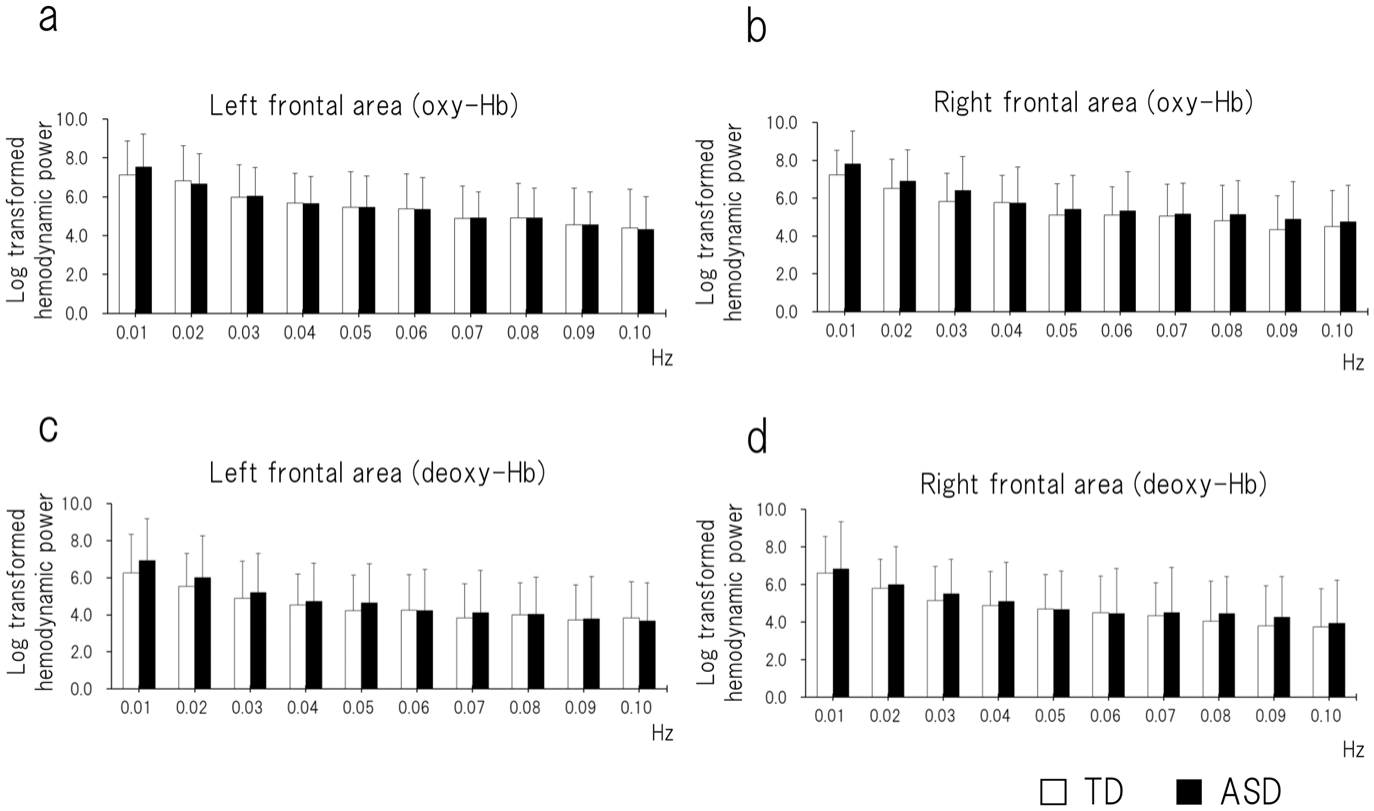
The absolute power value of the hemodynamic fluctuation. For ASD and TD children, the hemodynamic spectra were calculated with the fast Fourier transform from 0.01 to 0.10 Hz with a spectral resolution of 0.01 Hz. (a) Oxy-Hb in the left frontal area. (b) Oxy-Hb in the right frontal area. (c) Deoxy-Hb in the left frontal area. (d) Deoxy-Hb in the right frontal area. There was no significant difference between ASD and TD children at any frequency point. ASD, autism spectrum disorder; TD, typically developing.

There was no significant difference between ASD and TD children in any frequency point for oxy- or deoxy-Hb in either hemisphere (*P*>0.005; Fig. 2).

### Inter-hemispheric Hemodynamic Coherence

**Figure 3:**
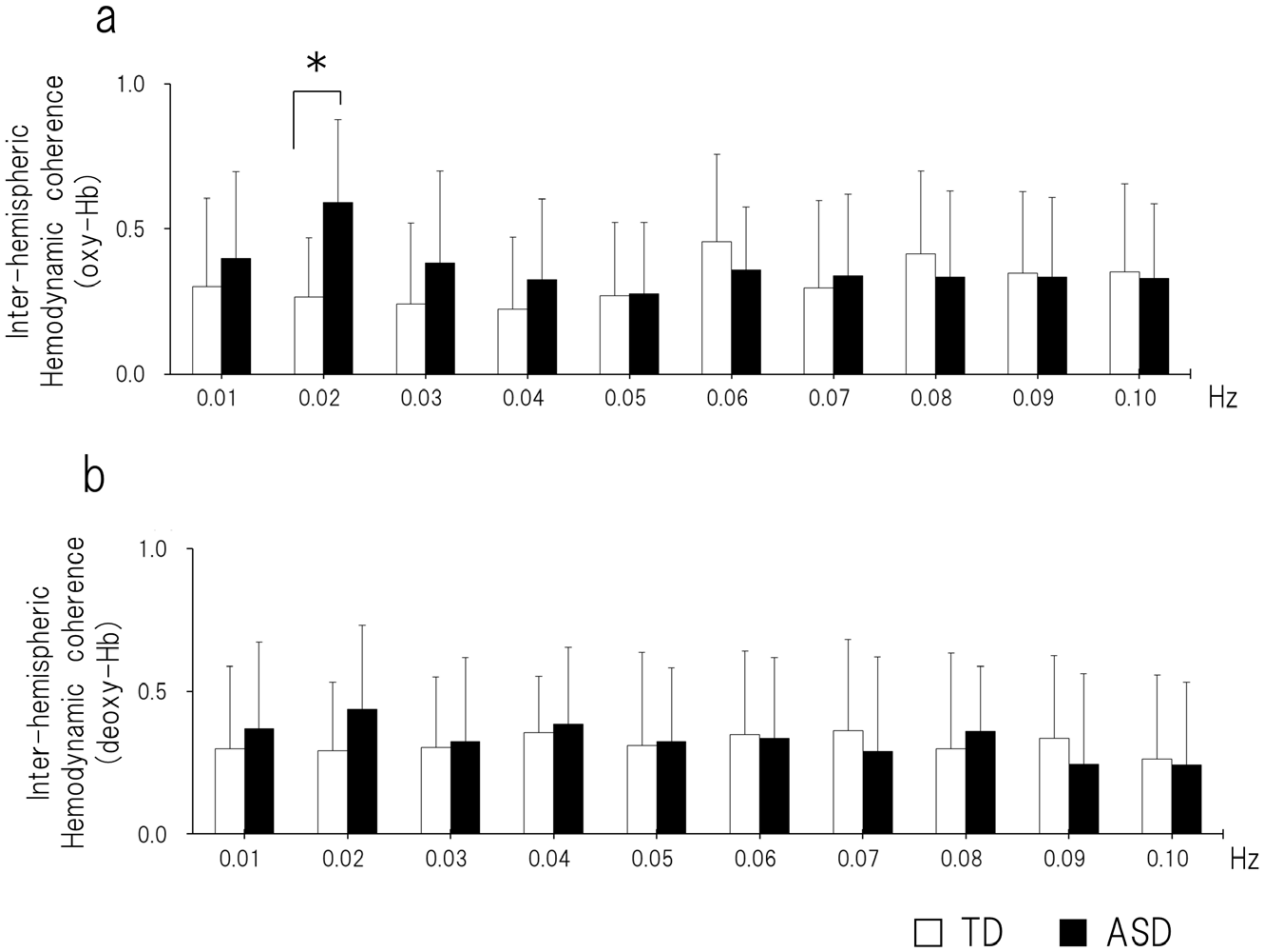
The inter-hemispheric coherence of the oxy-hemodynamic fluctuation. Coherence values were calculated from 0.01 to 0.10 Hz with a spectral resolution of 0.01 Hz. (a) In the oxy-hemodynamic fluctuation, there was a significant difference between ASD and TD children at 0.02 Hz (df = 28, t = −3.59, *P = *0.0012). (b) In the deoxy-hemodynamic fluctuation, there were no significant differences between the ASD and TD groups at any frequency point. ASD, autism spectrum disorder; TD, typically developing.

**Figure 4:**
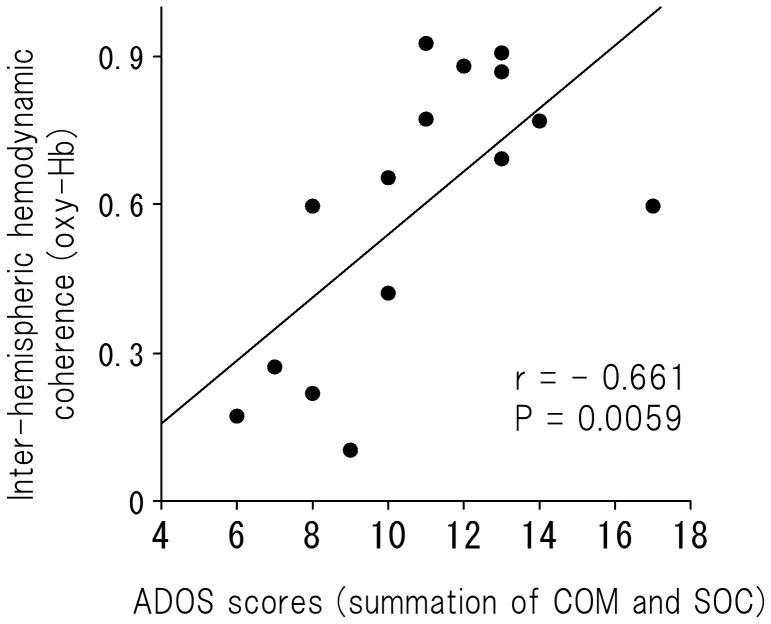
A scatter plot of the inter-hemispheric coherence (oxy-Hb) at 0.02 Hz hemodynamic fluctuation and the summation of COM and SOC scores on ADOS in children with ASD (r = −0.79, *P* = 0.0002). ASD, autism spectrum disorder; COM, communication domain; SOC, social interaction domain; ADOS, Autism Diagnostic Observational Schedule.

For oxy-Hb, there was a significant difference between ASD and TD children in the 0.02-Hz frequency (df = 28, t = −3.59, *P* = 0.0012; Fig. 3a). There was a significant positive correlation between the inter-hemispheric coherence in the 0.02 Hz frequency and social deficit for the ASD group (r = −0.661, *P* = 0.0059; Fig. 4). For deoxy-Hb, there were no significant differences between ASD and TD children at any frequency point (*P*>0.005; Fig. 3b).

To evaluate a potential effect of age and/or intelligence on the significant relationship found between the coherence in the 0.02-Hz frequency and the social deficit in young children with ASD, we employed multiple linear regression to predict the coherence in the 0.02-Hz frequency (i.e., the dependent variable) using social deficit, K-ABC achievement quotient and age as predictors (i.e., three independent variables). The significance level was set at p<0.05. The correlation coefficients among the three independent variables were 0.043 (between age and ADOS score), −0.192 (between ADOS score and K-ABC quotient) and 0.179 (between age and K-ABC quotient). In the multiple regression model, ADOS score was the significant predictor of the coherence at 0.02-Hz frequency (n = 15, β = 0.598, p = 0.0156), whereas age (n = 15, β = 0.002, p>0.05) and K-ABC quotient (n = 15, β = −0.327, p>0.05) did not reach statistical significance.

## Discussion

The current study provides initial evidence of higher bilateral aPFC connectivity in 0.02-Hz fluctuations in children with ASD. In addition, we found that subjects with lower social ability in the ASD group demonstrated this higher bilateral aPFC connectivity. NIRS methods have several advantages over fMRI methods. Compared to fMRI, NIRS methods are safer and less constrained, have less environmental noise, and are less sensitive to head motion. Especially under conscious conditions, NIRS is valuable for brain functional monitoring in young children [26]–[27], which has been difficult with fMRI. The 15 children with ASD in this study were 3 to 7 years old, which is the youngest ASD group that has been studied in terms of conscious-state hemodynamic brain connectivity.

A number of recent studies have suggested that autism is a disorder of cortical networks and that it is not associated with dysfunction in discrete cortical regions [28], [30]–[32], [40]–[50]. Although a cortical abnormality may result in functional impairment specific to that region, abnormal cortical connectivity results in impaired integration of the functions of the affected cortical regions. With recent developments in neuroimaging methods, such as functional connectivity magnetic resonance imaging (fcMRI) [51], the aberrant brain hemodynamic connectivity in ASD has been demonstrated in school-aged children (i.e., ages 8 and older) [31], [50], adolescents and adults [28], [30], [32], [40]–[48]. Although several previous studies have reported insufficient hemodynamic connectivity in the ASD cortex [25], [41]–[43], [47], recent studies on intrinsic brain connectivity networks computed by slow, spontaneous hemodynamic fluctuations (e.g., 0.01–0.10-Hz fluctuations) have uncovered regions of enhanced functional connectivity in ASD [28]–[32]. The present study found higher bilateral aPFC connectivity in 0.02-Hz fluctuations in young children with ASD under conscious conditions, and this higher connectivity was associated with lower social ability. Caution must be exercised in drawing definitive conclusions of extensive whole brain network from this regionally limited study. However, if a speculative discussion is permitted, this finding may be explained by immature networks that are characterized by fragmented patterns of resting-state hemodynamic networks [52]. A recent study using fcMRI investigated the resting-state networks in 5- to 8-year-old TD children under conscious conditions and demonstrated that components of the bilateral aPFC network were clearly identified and that the percentage of the explained variance in this network was the second-highest among 14 identifiable components [52]. In this previous study, this isolated and tight network within aPFC was regarded as an incomplete and fragmented pattern of a well-known network that has been observed in adult subjects (i.e., cingulo-opercular network) [53]. In addition, using multi-channel NIRS, a recent study on TD infants (neonates to 6 months old) demonstrated that higher inter-hemispheric connectivity in the prefrontal area is an immature characteristic of resting-state hemodynamic networks [54]. The immature or incomplete patterns of resting-state whole brain networks may contribute to the higher inter-hemispheric coherence in the anterior prefrontal area observed in children with ASD in the present study. Higher connectivity in aPFC reflects an aberrant strengthening of the relationship in the cortical regions and may be related to a deficit in selective brain organization that should have been achieved by synaptic pruning at young ages. Such higher connectivity supports the claim of abnormal pruning in children with autism [55]–[56].

From an anatomical perspective, using diffusion tensor imaging (DTI) to accurately estimate white matter, a number of recent studies have demonstrated the aberrant properties of frontal lobe fibers in patients with ASD. With regard to young children with ASD, results from the prefrontal area were controversial. Two recent studies have reported elevated fractional anisotropy (FA), which is a widely used measure of fiber integrity in white matter, in the prefrontal area. One study on 1- to 3-year-old children with ASD demonstrated a higher value of FA in the frontal area (e.g., forceps minor), which suggests an accelerated maturation of white matter [57]. Another study also demonstrated a higher value of FA in the forceps minor in 1- to 5-year-old children with ASD [58]. By contrast, three recent studies have reported reduced FA in the prefrontal area in young children with ASD [59]–[61]. On the other hand, with regard to older children and adults with ASD, reduced FA is consistently reported in several regions, including the genu of the corpus callosum [62]–[63]. The different developmental trajectory in the white matter fiber tract in children with ASD, which was reported in a recent longitudinal study [64], may explain this inconsistent result between young children and adults with ASD. That is, higher connectivity in the white matter fiber tract may be a temporal characteristic that is found only in young children with ASD. This temporal characteristic may be lost over time, resulting in consistently lower connectivity in older children and adults with ASD. The current finding of higher connectivity in the aPFC may be attributable to the anatomical higher connectivity in ASD that occurs only during young childhood.

One of the methodological shortcomings of NIRS is its relative treatment of values. The hemoglobin oxygenations that are measured with NIRS are relative to the initial measurement, and the oxygenations vary between subjects and within the head. However, the coherence function that was employed in the present study can overcome this methodological shortcoming because it is independent of the signal amplitude and determines the degree of the phase locking between activities that are recorded at different brain regions. Unlike the correlation coefficient, the coherence function can estimate the connectivity between two sets of time series data, regardless of the time lag between the coherent variations. In addition, the coherence value is a frequency and can determine the frequencies at which two sets of time series data are coherent.

There are several limitations to the present study. First, we measured the hemodynamic fluctuation only for the aPFC; thus, we could only achieve a limited connection and could not evaluate the intra-hemispheric connectivity along the anterior-posterior direction. Second, we employed a cross-sectional rather than a longitudinal design. Future research with a longitudinal design is necessary to provide a comprehensive understanding of developmental changes in hemodynamic brain connectivity in children with ASD. Third, a recent NIRS study reported the influence of skin blood flow on NIRS signals that were measured on the forehead during a verbal fluency task [65]. The authors suggested that frontopolar activation may represent a non-cortical physiological signal, which is an autonomic control, rather than cortical change. However, in the present study, power analyses in the right and left channels failed to demonstrate significant differences between children with ASD and TD children at any frequency points. It is implausible that the picture-card show enhanced autonomic skin blood flow fluctuations at any frequency band in children with ASD. Fourth, we could not evaluate how much the children attended to auditory or visual information in the picture-card show. Children with ASD might have attended to visual information rather than narrative sound information. Differences in modality-dependent preference may be reflected in brain functional connectivity. Although an attention-controlled condition is difficult for young children who are conscious, further study with such a condition will provide more reliable evidence. Fifth, to maintain the motion-noise-free condition for a 100-second continuous period without sedation, we employed a narrated picture-card show. In this condition, we could not evaluate the degree to which the picture-card show induced the coherent hemodynamic fluctuation between the right and the left aPFC. However, in the present study, power analyses for brain hemodynamic fluctuations failed to demonstrate significant differences between children with ASD and TD children at any frequency points. Therefore, it is implausible that the picture-card show enhanced brain hemodynamic fluctuations in the 0.02 Hz frequency points only for the children with ASD.
